# Crushing Characteristics of Coarse Aggregates for Asphalt Mixtures under Simulated Laboratory Compaction Loads and Repeated Traffic Loads

**DOI:** 10.3390/ma15175865

**Published:** 2022-08-25

**Authors:** Shijia Jiang, Hao Yu, Liantong Mo

**Affiliations:** 1 School of Material Science and Engineering, Wuhan University of Technology, Wuhan 430070, China; 2 State Key Laboratory of Silicate Materials for Architectures, Wuhan University of Technology, Wuhan 430070, China

**Keywords:** asphalt mixture, aggregate crushing, compaction loads, particle distribution, crushing mechanism

## Abstract

The crushing characteristics of coarse aggregates for asphalt concrete were investigated under static and dynamic aggregate crushing value tests (ACVTs). The effect of various compaction loads was also examined by using a Marshall hammer, gyratory compactor and steel roller. Six types of coarse aggregates were tested, including basalt aggregate, steel slag, limestone aggregate, marble aggregate, recycled concrete aggregate and slightly weathered limestone aggregate. Test results indicate that static ACVT failed to reflect the crushing behavior of coarse aggregates under traditional traffic and compaction loads. The type of aggregate strongly influenced the crushing resistance, independent of type of load. The compaction loads simulated by using a Marshall hammer, gyratory compactor and steel roller resulted in a high aggregate breakage ratio and can distinguish the coarse aggregates with high crushing susceptibility. The crushing resistance was evaluated by using various crushing parameters and the corresponding critical value of these parameters was established. Gyratory compactor compaction resulted in more serious aggregate crushing when compared to Marshall hammer and steel roller compaction. Finite element modelling results on roller compaction and Marshall hammer compaction are in agreement with the aggregate crushing results. The aggregate crushing mechanism was found to be controlled by the fracture mode; the contribution of the attrition and abrasion modes was relatively small. When coarse aggregates with low crushing resistance are considered for the use for asphalt mixture, proper compaction is proved to be vital to prevent excessive aggregate breakage during mixture preparation and construction.

## 1. Introduction

Asphalt mixture consists of coarse aggregates, fine aggregates, filler and asphalt binder. Coarse aggregates usually form the stone skeleton. Fine aggregates fill the voids of the coarse aggregate skeleton and asphaltic mastic that consists of bitumen, and the filler acts as the binder phase to bond all of the aggregates together as a whole. In such a structure, the coarse aggregate skeleton is the main load-carrying part and thus it is very important for the performance of asphalt mixture. In particular, the aggregate skeleton can significantly influence the rutting resistance of asphalt mixtures. A stable skeleton with good interlocking effects and load transfer ability is necessary to resist deformation during loading [1,2]. The strong and stable skeleton usually relies on high-quality aggregates. This is especially true for stone matrix asphalt and porous asphalt concrete. In these two types of asphalt mixtures, a stone-on-stone-like skeletal structure of gap-graded aggregate is applied to improve the performance of mixtures. In China, particular emphasis is placed on the high-temperature stability and rutting resistance for the design of dense asphalt mixture. As a result, a relatively high content of coarse aggregates, together with a low content of fine aggregates, is usually used for the design of aggregate-combined gradation. A typical example is that a very limited content of aggregate, between 2.36 mm and 4.75 mm, is used. This combination helps to obtain a relatively strong coarse aggregate skeleton without the disruption of fine fractions [3]. However, the stress distribution is expected to be unfavorable on stone-to-stone direct contact regions; concerns thus arise on aggregate crushing or breakage problems during laboratory compaction and under roller and traffic loads [4]. These concerns further strengthen the idea that only aggregates with high crushing resistance should be allowed for use. It also obviously restricts the application of some metamorphic rocks, and construction and demolition wastes (CDW) in asphalt mixtures [5,6,7].

The crushing characteristics of the aggregates are essential to ensure the stability of asphalt mixtures. The crushing resistance is usually evaluated quantitatively by using an aggregate crushing value test [8]. The tested aggregates are compacted and compressed in a cylinder by a plunger without lateral deformation. After loading to 400 kN, the aggregates are sieved, and the percentage of the crushed aggregates passing through a 2.36 mm sieve is determined as the aggregate crushing value (ACV). Important factors affecting ACV include mineral composition, particle shape, micromorphology, size of aggregate, load rate and level. Furthermore, a single-particle crushing test and a multi-particle confined compression test were proposed for better insight into the breakage behavior of aggregate particles. It was found that the contact location distribution strongly influenced the crushing of granular materials and the particle strength increased with the number of contacts [9]. The particle alignment increased the concentrated local failure leading to breakage and affected the development of force chains during failure [10]. Liu and Qin investigated the particle breakage of coarse aggregates based on large-scale triaxial tests. It was reported that the particle breakage index increased with the increase in confining pressures. The increase in particle breakage resulted in a decrease in the strength of coarse aggregates [11]. Cai and Qiu investigated the compaction performance and particle breakage characteristics of crushed stone as the aggregate of permeable roads. It was found that particle crushing commonly fell into four categories, including complete crushing, complete rupture, partial damage and surface grinding. Single-particle-size samples were subject to stronger particle breakage than the mixed-particle-size samples [12]. Zhang and Tang investigated the crushing mechanism of recycled aggregates by using a single-particle crushing test, multi-particle crushing test and discrete element modeling. It was found that the volume proportion of the gravel significantly affected the mechanical behavior of the mixed aggregates, and 75% was the critical threshold of the gravel component [13]. Several studies also reported that the phenomena of aggregate crushing and breakage happened during the laboratory compaction of asphalt mixtures by using a Marshall hammer and gyratory compactor [6,14,15,16]. The latter seemed to be promising in estimating the degradation behavior of the aggregate skeleton [7,17]. An obvious change in aggregate gradation was reported after mixture compaction, especially that containing poor aggregates. Some of the coarse aggregates were crushed and became a portion of fine fractions. A laboratory-accelerated heavy-loading test indicated that the coarser aggregates with a size of 9.5–16 mm were more likely to be crushed, and thus weakened the stability of the aggregate skeleton structure under cyclic loading [18]. Field materials extracted from stone matrix asphalt (SMA) pavements indicated that asphalt mixture obtained from traffic lanes had finer gradation than those from non-traffic lanes after long-time service, implying degradation of the aggregate skeleton [12].

Asphalt concrete is subjected to complex static and dynamic loading combinations induced by laboratory compaction, field construction compaction and repeated traffic loads, which may result in the breakage of fragile aggregates. Previous studies mainly focused on the effect of static loads on aggregate crushing, and limited research has been conducted on the effect of dynamic traffic loads on aggregate breakage during road service. When facing a lack of high-quality aggregate, the use of low-quality aggregates, which are locally abundant, becomes necessary for cost saving. However, the risk of aggregate crushing during laboratory compaction, field construction compaction and repeated traffic loads becomes a big concern. The conventional aggregate crushing value test (ACVT) applies a load of 400 kN, which results in a compressive stress of 22.65 MPa. This stress level is believed to be far higher than those to which coarse aggregates are subjected under actual compaction and traffic loads. The static load of ACVT may not well reflect the crushing behavior of aggregates under these real loads. The selection of coarse aggregate based on ACVT has its limits.

This study aims to investigate the crushing behavior of coarse aggregates for asphalt mixtures under simulated laboratory compaction, field construction compaction and repeated traffic loads. The laboratory compaction loads were simulated by using a traditional Marshall Hammer, gyratory compactor and steel roller. The effect of repeated traffic loads on aggregate crushing was investigated by using a dynamic ACVT with 100,000 loading cycles. A wide range of types of aggregates with different crushing susceptibility was considered to obtain fundamental insight into aggregate crushing. The types of coarse aggregates included basalt aggregate, steel slag, limestone aggregate, marble aggregate, recycled concrete aggregate and slightly weathered aggregate. The crushing mode was investigated by the particle size distribution of crushed aggregates after testing. Particle breakage potential was proposed to discuss the aggregate crushing mechanisms under various compression loads. The accumulative permanent strain and resilient modulus were discussed under cyclic loads and their developments over loading cycles were modelled. Finite element modelling was conducted to investigate the effect of roller compaction in field and conventional laboratory Marshall compaction on the risk of aggregate breakage. Finally, fundamental insights into the crushing characteristics of various coarse aggregates for asphalt mixtures were obtained under various compaction loads and repeated traffic loads. This is useful to guide the application of coarse aggregates with high crushing susceptibility in the field of asphalt concrete.

## 2. Materials and Methods

### 2.1. Materials

Six different types of coarse aggregate, including basalt aggregate, steel slag, limestone aggregate, marble aggregate, recycled concrete aggregate and slightly weathered limestone aggregate, were used in this study. Some basic properties, including apparent relative density, water absorption, aggregate crushing value and polished stone value, are shown in Table 1. These tests were performed according to the Chinese specification of Test Methods of Aggregate for Highway Engineering [8]. Among these types of aggregate, basalt aggregate is commonly used for the surface wearing course of asphalt pavements in China because it has a high polished stone value that reflects an excellent resistance to the polishing action of vehicle tires. Steel slag aggregate was obtained from Wuyang Iron and Steel Holding Group, Henan province, China, by means of a hot stuffing process. Limestone aggregate, which has a relatively low polished stone value, is widely used for the intermediate and base course layers of asphalt pavements. Other aggregates, including marble aggregate, recycled concrete aggregate and slightly weathered limestone aggregate, tend to have a high crushing value and thus have a relatively poor crushing resistance during compaction and service. These types of aggregate are usually recommended for the base course layer of asphalt pavements. According to the Technical Specifications for Construction of Highway Asphalt Pavements, the limit of the aggregate crushing value (ACV) is dependent on the grade of highway, as well as the layer where the aggregate is used [19]. For the wearing course of high-grade highway, the limit of the ACV is 26% and, for the intermediate and base course layers, the limit of the ACV is a higher value of 28%. For low-grade highway, the limit of the aggregate crushing value is designed as 30%, independent of types of layers.

### 2.2. Test Methods

**Figure 1:**
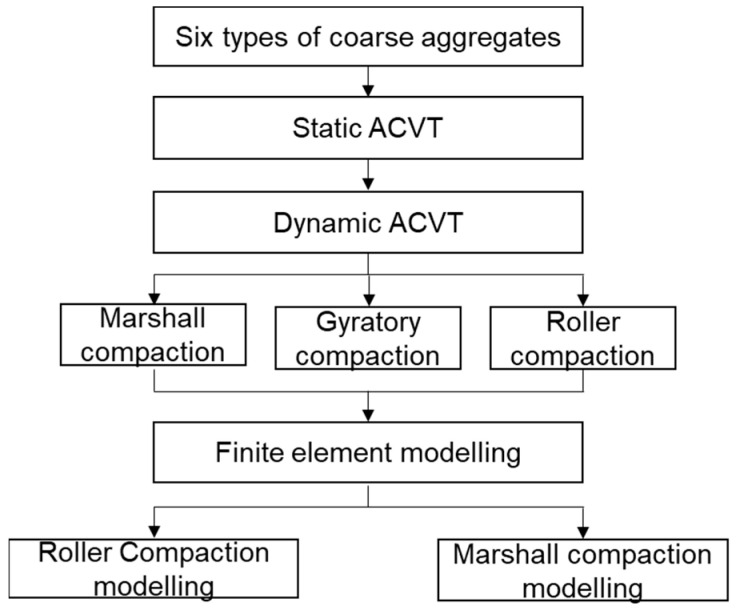
Flow chart of the study content.

Figure 1 shows the flow chart of the study content. Six types of coarse aggregate were subjected to static and dynamic ACVTs. After that, various compaction tests were carried out with a Marshall hammer, gyratory compactor and steel roller. Finally, finite element modelling on steel roller and Marshall compaction tests was conducted.

#### 2.2.1. Aggregate Crushing Value Test (ACVT) under Static Load

In this study, static ACVTs on various types of coarse aggregates, as mentioned above, were done to obtain insight into the crushing resistance under a linearly applied compressive load of 400 kN within 10 min. A single-size aggregate between 9.5 mm and 13.2 mm was selected for each static ACVT. About 3 kg of coarse aggregates was carefully placed to fill the measuring cylinder with a diameter of 150 mm in three layers. The load was applied at a rate of 40 kN/min until it reached 400 kN. After the load was released, the crushed aggregate was taken out of the cylinder and sieved through a 2.36 mm sieve. The percentage of the crushed aggregates passing through the 2.36 mm sieve was determined as the aggregate crushing value (ACV). In order to investigate the particle distribution after the static ACVT, a full sieve analysis of the crushed aggregate was carried out. In this case, sieves of 0.075 mm, 0.15 mm, 0.3 mm, 0.6 mm, 1.18 mm, 2.36 mm, 4.75 mm, 9.5 mm and 13.2 mm were involved. Insight into the aggregate breakage mode was discussed according to particle distribution characteristics.

#### 2.2.2. Dynamic ACVT under Cyclic Loading

**Figure 2:**
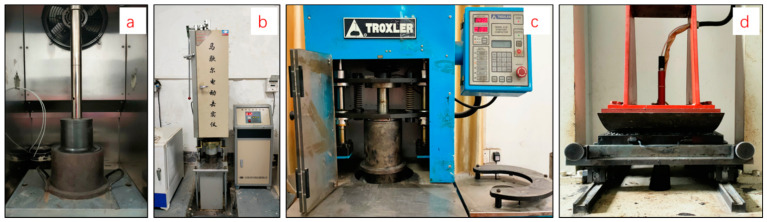
Test setup for aggregate crushing resistance analysis ((**a**): dynamic ACVT; (**b**): Marshall hammer compaction; (**c**): gyratory compaction; (**d**): steel roller compaction).

The aggregate used in roads and pavement construction must be strong enough to withstand crushing under roller and traffic-induced loads. In this case, the static ACVT does not reflect well the effect of cyclic loading of rollers and traffic. Furthermore, in the static ACVT, a compressive stress of about 22.65 MPa is applied. This stress level is believed to be very high when compared with that which is induced by actual roller and traffic loads. Dynamic ACVTs under cyclic loading with a relatively low stress level are thus of interest. For this reason, a half-sinusoidal wave of compressive stress was applied for cyclic loading. In order to reflect the roller–road and tire–road interaction force, the stress amplitude was considered at three levels, including 0.7 MPa, 1.4 MPa and 2.1 MPa. Among these three stress levels, 0.7 MPa represents the standard tire–road contact stress. Dynamic ACVTs under cyclic loading were carried out by using a UTM-130 test machine and the test setup is shown in Figure 2. The loading frequency was 2 Hz with a half-sine compressive stress and, in total, 100,000 cycles were applied for each test. For each load cycle, the loading time was 0.1 s and followed by a rest time of 0.4 s. The accumulative permanent vertical strain and resilient modulus was determined to investigate the mechanical properties. After the dynamic ACVT, the tested aggregate was taken out and the sieve analysis performed for the particle distribution.

#### 2.2.3. Marshall Hammer Compaction

The Marshall mix design and compaction method are the primary method for asphalt mix design around the world. The bituminous specimens are prepared and compacted using Marshall impact hammers, as indicated in Figure 2. The compressive impact loads are delivered by the compaction hammer of a 4.5 kg weight after a free fall of 457 mm. The designed number of impact blows are selected according to the design traffic category of the asphalt mixture. For light traffic, 50 blows for each side are usually applied, while 75 blows for each side are applied for heavy traffic. The impact loads induced by the Marshall hammer are expected to result in aggregate crushing and breakage, and thus affect the volumetric and mechanical properties of the compacted asphalt concrete. This is especially true for the application of fragile aggregates. In this study, a Marshall impact hammer was applied to investigate the crushing behavior of coarse aggregates without the addition of fine aggregate, filler and bitumen. About 800 g of coarse aggregate with a single size of 9.5–13.2 mm was carefully put into the steel mold with an inside diameter of 101.6 mm and a height of 75 mm. The sample was compacted by 100 impact blows on the top side. After compaction, the tested aggregate was taken out from the mold and the sieve analysis carried out to obtain the particle size distribution.

#### 2.2.4. Gyratory Compaction

Compared to Marshall compactors, gyratory compactors are able to simulate the aggregate particle orientation during actual field compaction and thus is considered to be a better method of laboratory compaction. Compaction in a gyratory compactor was reported to be highly sensitive to the compaction pressure, the angle of gyration and the number of gyrations [20]. In this study, a gyratory compactor, as indicated in Figure 2, was used to compact coarse aggregate samples instead of hot asphalt mixture samples. The gyratory compaction test was carried out with a compaction pressure of 600 kPa, a gyration rate of 30 revolutions per minute and a constant angle of gyration of 1.25 degrees. The number of gyrations was selected as 205 gyrations to reflect the effects of heavy traffic. About 3 kg of coarse aggregate with a single size of 9.5–13.2 mm was carefully put into the steel mold with an inside diameter of 150 mm in three layers. After gyratory compaction, the tested aggregate was taken out from the mold and the sieve analysis carried out.

#### 2.2.5. Steel Roller Compaction

Asphalt mixture specimens can also be prepared in the laboratory by the wheel-rolling method. These specimens are commonly used for the high-temperature rutting test and other mechanical tests. In this study, a steel roller compactor, as indicated in Figure 2, was used to compact the unbound coarse-aggregate test sample, instead of a hot asphalt mixture. The roller had a diameter of 500 mm and a width of 300 mm. The roller compaction was applied by a deadweight of 9000 N, thus introduced a static linear load of 300 N/cm. The steel mold used was 300 mm in length, 300 mm in width and 50 mm in depth. About 7 kg of coarse aggregate with a single size of 9.5–13.2 mm was put into the mold for roller compaction. According to Standard Test Methods of Bitumen and Bituminous Mixtures for Highway Engineering, the number of rolling can be varied between 24 and 48 passages, depending on the type of mixture and the desired density of asphalt concrete test specimens. In this study, 36 passages were selected for roller compaction, which represented the typical compaction level for asphalt concrete slab specimens. Similarly, the tested aggregate was taken out from the mold and the sieve analysis carried out after roller compaction.

## 3. Results and Discussion

### 3.1. Static ACVT Results

Table 2 gives a summary of aggregate particle distribution after the static ACVTs for various types of aggregate. It should be noted that the tested aggregate was a single size between 9.5 mm and 13.2 mm for each static ACVT. After testing, a wide range of aggregate particle distribution was observed, as listed in Table 2. Most of the aggregate was crushed and became a portion of finer fractions. The crushing characteristics strongly depended on type of aggregate. In general, basalt aggregate showed a strong crushing resistance, with 53.54% of the aggregate retained with a sieve size of 9.5 mm. The worst case was found with recycled concrete aggregate, with only 11.75% of the aggregate retained with a sieve size of 9.5 mm.

**Figure 3:**
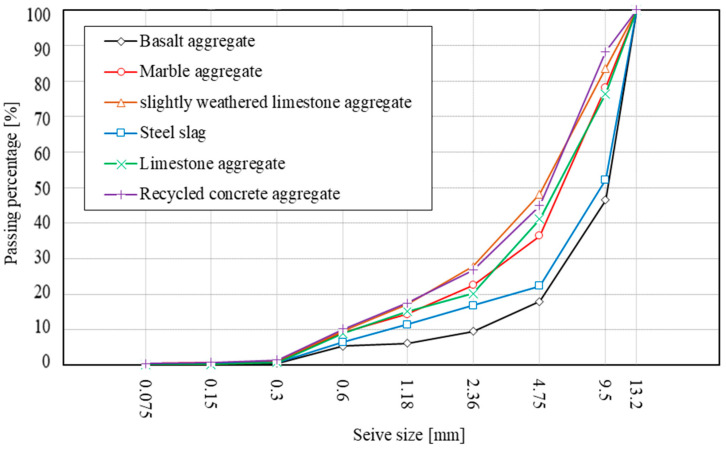
Aggregate grading curves after static ACVTs.

Figure 3 shows the aggregate grading curves after static ACVTs for various types of aggregate. Basalt aggregate had a relatively lower passing percentage through a wide sieve size, which indicates a strong resistance to breakage under compression. Steel slag showed a comparable curve with that of basalt aggregate. Limestone and marble aggregates showed a similar grading after static ACVT, with a slight difference in the passing percentage with a 4.75 mm sieve. The unfavorable types of aggregate were found to be recycled concrete aggregate and slightly weathered limestone aggregate. As expected, these two types of aggregate exhibited a curve of high passing percentage. Since a single-size aggregate between 9.5 mm and 13.2 mm was tested, the passing percentage of the 9.5 mm sieve can be defined as the breakage ratio, which indicated the percentage of aggregate that was crushed and became smaller than 9.5 mm. In this case, basalt aggregate had a breakage ratio of 46.5%, followed by steel slag with a breakage ratio of 52.20%, while limestone and marble aggregates had a breakage ratio of 76.4% and 78.2%, respectively. The unfavorable aggregates, recycled concrete aggregate and slightly weathered limestone aggregate, have a breakage ratio of 88.25.4% and 83.50%, respectively. This indicates the strong crushing resistance of basalt aggregate and steel slag compared with other types of aggregate.

In order to investigate the change of aggregate size before and after testing, the aggregate equivalent size was calculated using the following equations:

(1) $$d\;or\;d^{\prime }=\sum_{n=1}^{\infty }P_{i}\times d_{i}$$

(2) $$\Delta d=d-d^{\prime }$$

where:

d = Aggregate equivalent size before testing;
$d^{\prime }$ = Aggregate equivalent size after testing;
Δd = Change of equivalent aggregate size;
$d_{i}$ = Aggregate equivalent size at i-th sieve;
$P_{i}$ = Percentage of aggregate with a single size of $d_{i}$.

**Figure 4:**
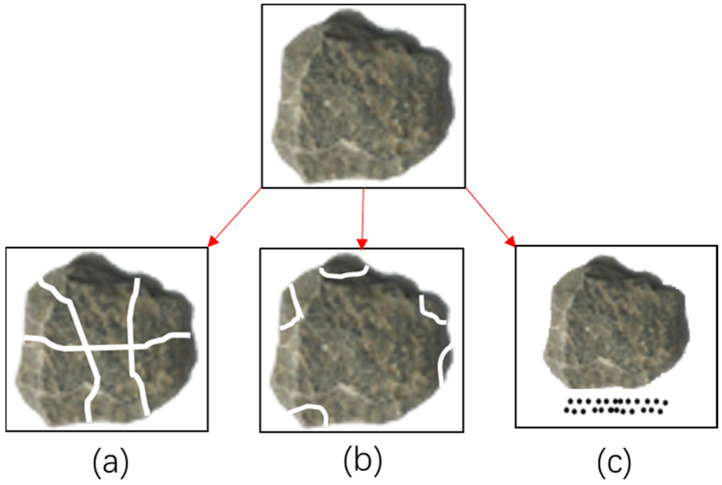
Schematic diagram of aggregate breakage modes ((**a**): fracture mode; (**b**): attrition mode; (**c**): abrasion mode).

As shown in Figure 4, the aggregate breakage can be divided into three different modes, including fracture, attrition and abrasion [21]. Each breakage mode has its distinctive particle size distribution. Among these modes, the fracture mode tends to break aggregate into multiple coarse parts, while the attrition mode is likely to result in small parts due to local fragmentation. The abrasion mode leads to the finest parts due to surface grinding [22,23].

In order to give a better assessment of particle breakage over the whole range of particle sizes, relative breakage potential was proposed based on an ultimate size of 0.075 mm for the possible aggregate crushing [24]. The relative breakage potential (RBP) was defined as follows:

RBP = A_0_/A_1_ × 100% (3)

where:

A_0_ = the total breakage defined as the area between the initial and crushed aggregate grading curves;
A_1_ = the potential for breakage defined as the area between the initial and ultimate aggregate grading curves.

**Figure 5:**
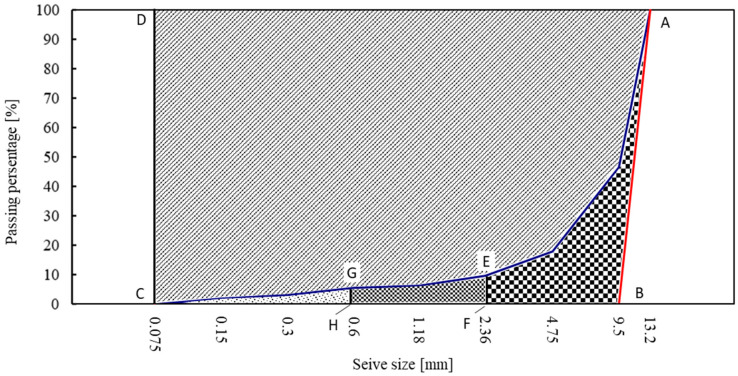
Schematic diagram of the determination of aggregate breakage potential.

As illustrated in Figure 5, the sieve size was plotted in log scale as the horizontal coordinate. The initial aggregate grading curve was indicated by the straight line of A–B because of the single-size aggregate between 9.5 mm and 13.2 mm. The crushed aggregate grading curve was presented by the curve of A–E–G–C. The total breakage A_0_ was determined by the total area enclosed by A–E–G–C–B. The potential for breakage A_1_ was defined by the area enclosed by A–B–C–D. In order to investigate the effect of the breakage mode on relative breakage potential, the total breakage was further sub-divided into three categories: the coarse (2.36–13.2 mm) fraction, the small (0.6–2.36 mm)-size fraction and the fine (0.075–0.6 mm) fraction, which are indicated by the area of A–B–F–E, F–E–G–H and H–G–C, respectively.

Table 3 gives the results of the aggregate crushing characteristics under static ACVTs. For the purpose of comparison, the breakage ratio, ACV, change of aggregate size and relative breakage potential are listed in Table 3. Since a single-size aggregate between 9.5 mm and 13.2 mm was used, the aggregate equivalent size before test was determined by the average of 9.5 mm and 13.2 mm, that is 11.35 mm. After testing, the aggregate equivalent size was reduced to 8.49 mm, 7.91 mm, 6.16 mm, 5.33 mm, 6.11 mm and 5.40 mm for basalt, steel slag, limestone, marble, recycled concrete aggregate and slightly weathered limestone aggregate, respectively. The corresponding change of aggregate equivalent size ranged from 2.86 mm to 6.02 mm. This indicated that the coarse aggregates were crushed into finer aggregate and the crushing behavior was dependent on the type of aggregate.

As indicated in Table 3, The relative breakage potential of coarse, small and fine fractions was also determined after static ACVTs. In general, basalt aggregate had the lowest relative breakage potential, followed by steel slag, limestone, recycled concrete aggregate and slightly weathered limestone aggregate; marble aggregate tended to have the highest relative breakage potential. However, the difference between marble aggregate and slightly weathered limestone aggregate was very limited. A similar phenomenon was also found between limestone and recycled concrete aggregate. Among the contributions of coarse (2.36–13.2 mm) fraction, small (0.6–2.36 mm)-size fraction and fine (0.075–0.6 mm) fraction, the coarse fraction had the greatest influence on relative breakage potential, indicating the fracture mode was dominant for aggregate breakage under static ACVTs. Effects of the attrition and abrasion modes was relatively small. This was also in agreement with the test data obtained from the breakage ratio and ACV.

**Figure 6:**
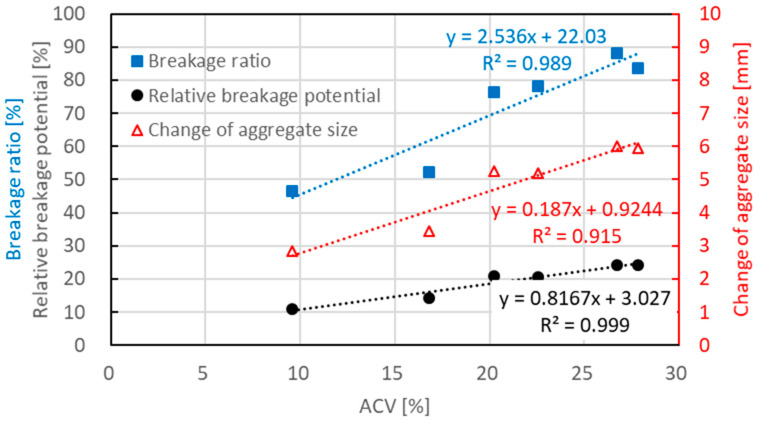
Relations of breakage ratio, change of aggregate size and relative breakage potential to ACV.

Figure 6 shows the relations of breakage ratio, change of aggregate size and relative breakage potential to ACV. Linear relations with ACV were observed for these three breakage parameters. Higher ACV tended to result in a higher breakage ratio, change of aggregate size and relative breakage potential. It strongly indicated that these crushing indexes had good correlations and each one can well explain the aggregate crushing resistance.

### 3.2. Dynamic ACVT Results

#### 3.2.1. Resilient Modulus and Accumulative Permanent Strain

**Figure 7:**
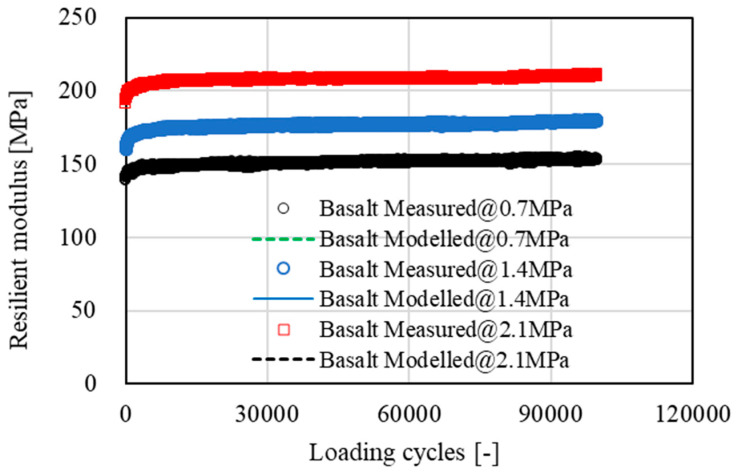
Development of resilient modulus of basalt aggregate under cyclic loading.

**Figure 8:**
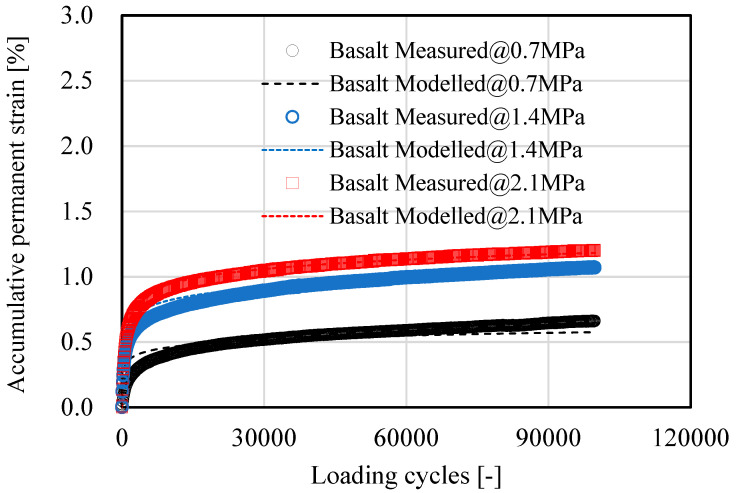
Development of accumulative permanent strain of basalt aggregate under cyclic loading.

**Figure 9:**
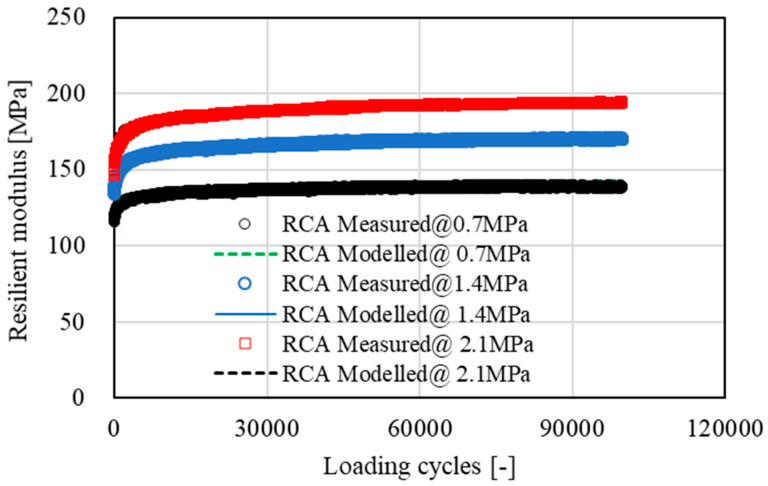
Development of resilient modulus of recycled concrete aggregate (RCA) under cyclic loading.

**Figure 10:**
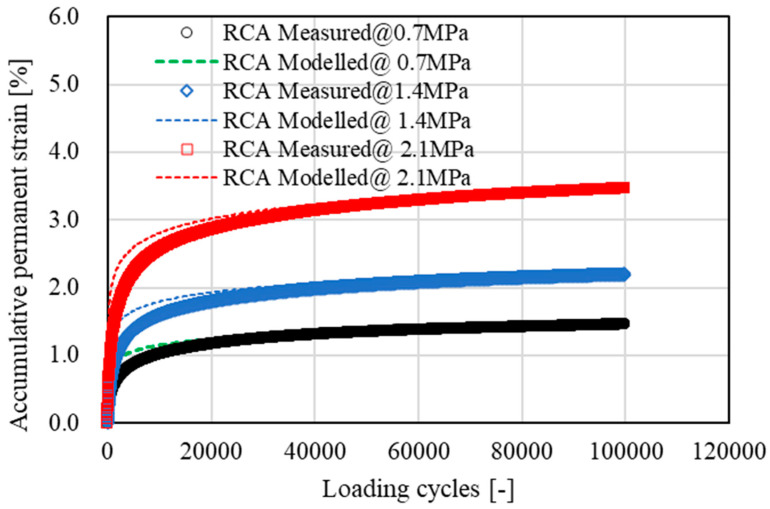
Development of accumulative permanent strain of recycled concrete aggregate (RCA) under cyclic loading.

Figure 7, Figure 8, Figure 9 and Figure 10 show the development of resilient modulus and accumulative permanent strain of basalt aggregate and recycled concrete aggregate under cyclic loading. Similar test results were also obtained on other types of aggregate. For the purpose of simplicity, only the data obtained from basalt aggregate and recycled concrete aggregate were plotted for illustration. Since all types of aggregate exhibited similar development tendencies of resilient modulus and accumulative permanent strain under cyclic loading, the following two equations were used to fit the obtained test results:

(4) $$E=A\times \ln\left(N\right)+E_{0}$$

(5) $$\varepsilon_{p}=C\times \ln\left(N\right)$$

where:

E = resilient modulus;
A, C = the model fitting parameter;
N = the number of loading cycles;
*E*_0_ = the initial resilient modulus;
$\varepsilon_{p}$ = the accumulative permanent strain.

As indicated in Figure 7, Figure 8, Figure 9 and Figure 10, the proposed equations fit the obtained test data well. Some of the fitting lines were completely covered by the data points. The fitting results of the related model parameters are listed in Table 4. For each type of aggregate, three compressive stress levels were carried out for dynamic ACVTs. As indicated in Figure 7 and Figure 9, the resilient modulus increased rapidly at the early state. Further increased loading cycles led to a steady increase in resilient modulus. Increasing stress levels resulted in an obvious increase in resilient modulus. For instance, the resilient modulus of basalt aggregate increased from 155 MPa to 180 MPa and 210 MPa as the corresponding stress level increased from 0.7 MPa to 1.4 MPa and 2.1 MPa, respectively. This indicated a strong stress dependency of the resilient modulus. A similar phenomenon was also observed for recycled concrete aggregate. The resilient modulus increased from 138 MPa to 170 MPa and 198 MPa as the corresponding stress level increased.

With respect to accumulative permanent strain, as indicated in Figure 8 and Figure 10, a rapid increase was observed at the beginning state. As loading cycle increased, a knee point appeared and followed a steady growth. The accumulative permanent strain exhibited an obvious stress dependency. For example, after 10,000 loading cycles, the accumulative permanent strain of basalt aggregate was 0.65%, 1.08% and 1.21% for the corresponding stress levels of 0.7 MPa to 1.4 MPa and 2.1 MPa, respectively. At the same stress level, a higher accumulative permanent strain was found for recycled concrete aggregate under cyclic loading.

**Figure 11:**
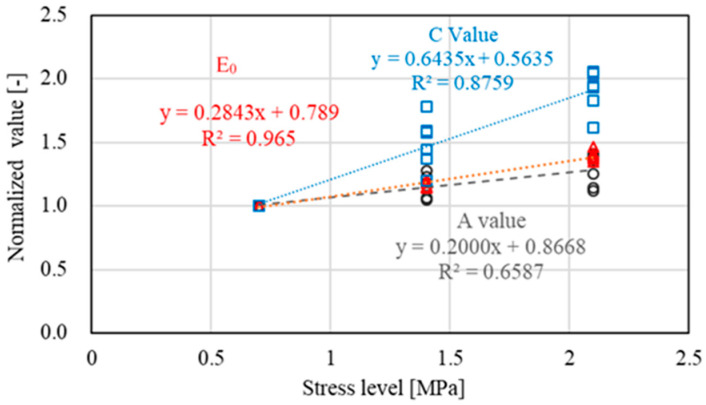
Stress dependence of model parameters based on normalized analysis.

Figure 11 shows the stress dependence of the model parameters, including A, E_0_ and C, based on normalized analysis using the value at 0.7 MPa as a reference. For each type of aggregate, the value at 0.7 MPa was first normalized as 1, and the remaining values at 1.4 MPa and 2.1 MPa were calculated by the relative ratio using the corresponding value divided by the one at 0.7 MPa. As indicated, E_0_ and C showed a stronger stress dependency compared with the value of A. This further proved that higher stress levels could lead to a higher E_0_ and permanent strain of unbound coarse aggregate. This stress dependency seemed to be independent of the tested type of aggregate.

#### 3.2.2. Aggregate Crushing Characteristics under Dynamic ACVTs

Table 5 gives the particle size distribution after dynamic ACVTs for various types of aggregate with a single size of 9.5–13.2 mm. As observed in Table 5, more than 85% of aggregates were retained between 9.5 mm and 13.2 mm after testing. This meant that less than 15% of single-size aggregates were crushed after 100,000 loading cycles. Among the crushed aggregates, the coarse (2.36–13.2 mm) fraction was more than 98%, the small (0.6–2.36 mm) fraction was less than 1% and the fine (0–0.6 mm) fraction was less than 0.5%. Compared with traditional static ACVTs, the aggregate crushing mode and extent were obviously different under dynamic ACVTs. The main reason can be attributed to the distinct difference of applied loading level. In static ACVTs a stress level of 22 MPa was applied, while in dynamic ACVTs, a stress level ranging from 0.7 MPa to 2.1 MPa was applied in this study. The analysis of aggregate particle distribution also indicated 100,000 repeated loading cycles did not result in a marked aggregate crushing. As the applied stress level increased, the percent retained between 9.5 mm and 13.2 mm tended to decline. This tendency to decline was slight for basalt aggregate, while it became significant for recycled concrete aggregate and slightly weathered limestone aggregate. Steel slag showed a comparable crushing resistance with basalt aggregate under dynamic loads. When compared with the percent retained at various aggregate sizes, it was found that most of the crushed aggregate became a substantial portion of 4.75–9.5 mm aggregate. The contribution of coarse (2.36–9.5 mm) fraction, small (0.6–2.36 mm) fraction and fine (0–0.6 mm) fraction remained within 0.13–10.87%, 0.02–1.1% and 0.07–0.58%, respectively, under dynamic ACVTs. This indicated that the crushing mechanism was prominent for the fracture mode due to a limited amount of fragile aggregate.

Table 6 gives the results of aggregate crushing characteristics under dynamic ACVTs. It indicates that the breakage ratio, Δd, and relative breakage potential all increased with the increased stress level. However, compared with the data listed in Table 3, the changes of these three indexes were found to be relatively limited. For example, the breakage ratio ranged from 0.22% to 12.54% for dynamic ACVTs, while for static ACVTs, it ranged from 46.46% to 88.25%. With respect to Δd, the range was within 0.02~0.67 mm and 2.86~6.02 mm for dynamic and static ACVTs, respectively. Similarly, the relative breakage potential was within 0.08~2.18% and 10.91~24.19% for dynamic and static ACVTs, respectively. As indicated above, the results obtained from dynamic and standard ACVTs have an order of magnitude difference. This implied that the crushing behavior under static loads with a high stress level failed to reflect well that under dynamic loads with relatively low stress levels.

### 3.3. Aggregate Crushing Characteristics under Marshall Hammer Compaction

Table 7 and Table 8 give the aggregate particle distribution and crushing characteristics after Marshal hammer compaction. It can be seen that the percent retained between 9.5 mm and 13.2 mm varied from 64.05% to 87.38%. This indicated that approximately 12.62~35.95% of the single-size aggregates were crushed after 100 impact blows induced by a Marshal hammer. In general, basalt aggregate and steel slag had a fairly good crushing resistance, with a breakage ratio of about 12.62~18.14%, while the remaining four types of aggregate showed a relatively larger breakage ratio between 30% and 35%. The aggregate tended to be crushed and mainly became a portion of 4.75–9.5 mm fraction and 2.36–4.75 mm fraction. The contribution of coarse (2.36–9.5 mm) fraction, small (0.6–2.36 mm) fraction and fine (0–0.6 mm) fraction remained within 11.34–29.09%, 0.61–5.13% and 0.69–2.37%, respectively.

As listed in Table 8, the range of Δd. was within 0.64~2.13 mm and the relative breakage potential was within 2.07~7.45% for Marshall hammer compaction. However, the data in Table 3 show static ACVTs had the range of Δd. within 2.86~6.02 mm and a relative breakage potential within 10.91~24.19%. The difference indicated that the effects of static ACVTs on aggregate crushing was 3–4 times stronger than that of Marshall hammer compaction. With respect to the relative breakage potential of coarse, small and fine fractions after Marshall hammer compaction, it was found that the relative breakage potential of coarse fractions occupied a predominant position. The percent retained at various aggregate sizes combined, it was found that the fracture mode was prominent for Marshall hammer compaction.

### 3.4. Aggregate Crushing Characteristics under Gyratory Compaction

Table 9 and Table 10 give the aggregate particle distribution and crushing characteristics after gyratory compaction for various types of aggregate. It can be seen that the breakage ratio was within a range of 11.24~48.20% for various types of aggregate after 205 gyrations. Among these aggregates, both basalt aggregate and steel slag showed a fairly good crushing resistance, with a breakage ratio of about 11.24~14.24%, while the remaining four types of aggregate showed a relatively larger breakage ratio between 30% and 50%. Similarly, the aggregate tended to be crushed and mainly became a portion of 4.75–9.5 mm fraction and 2.36–4.75 mm fraction. The contribution of coarse (2.36–9.5 mm) fraction, small (0.6–2.36 mm) fraction and fine (0–0.6 mm) fraction remained within 9.66–38.00%, 0.68–5.46% and 0.90–4.80%, respectively.

As listed in Table 10, the range of Δd was within 0.60~2.97 mm and the relative breakage potential was within 2.11~11.26% for gyratory compaction. When compared to static ACVTs, a distinct difference was also observed, which also indicated that the effect of static ACVTs on aggregate crushing was 2–4 times stronger than that of static gyratory compaction. The results of gyratory compaction were generally comparable to those of Marshall hammer compaction. With respect to the relative breakage potential of coarse, small and fine fractions after gyratory compaction, it was also found that the relative breakage potential of coarse fractions was predominant compared with the other two relative breakage potentials. This indicated that the fracture mode was prominent for gyratory compaction.

### 3.5. Aggregate Crushing Characteristics under Roller Compaction

Table 11 and Table 12 show the aggregate particle distribution and crushing characteristic under roller compaction for various types of aggregate. Similarly, the coarse aggregates were crushed and mainly became a portion of 4.75–9.5 mm fraction and 2.36–4.75 mm fraction. The contribution of coarse (2.36–9.5 mm) fraction, small (0.6–2.36 mm) fraction and fine (0–0.6 mm) fraction remained within 10.00–30.80%, 0.62–2.10% and 1.05–2.80%, respectively. It can be seen that the breakage ratio was within a range of 11.67~35.70% for various types of aggregate. Among these aggregates, basalt aggregate and steel slag had a fairly good crushing resistance, with a breakage ratio of about 11.67~14.29%, while the remaining four types of aggregate showed a relatively larger breakage ratio between 20.33% and 35.70%.

As listed in Table 12, the range of Δd was within 0.63~1.93 mm and the relative breakage potential was within 2.26~6.76% for roller compaction. When compared to static ACVTs, a distinct difference was also observed, which indicated that the effect of static ACVTs on aggregate crushing was 3–4 times stronger than that of roller compaction. However, the results of roller compaction were comparable to those of Marshall hammer and gyratory compaction. This indicated that the traditional three compaction methods tended to result in similar aggregate crushing. With respect to the relative breakage potential of coarse, small and fine fractions after roller compaction, it was found that coarse fraction was predominant, while the small and fine fractions were relatively limited. This also indicated that the fracture mode accounted for the cause of aggregate crushing under roller compaction.

### 3.6. Comprehensive Analysis of Aggregate Crushing Characteristics

**Figure 12:**
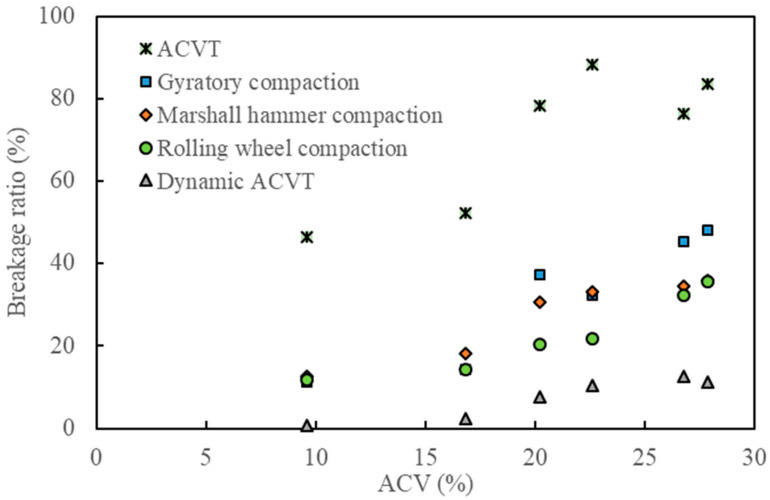
Effect of ACV on the breakage ratio under various loading methods.

**Figure 13:**
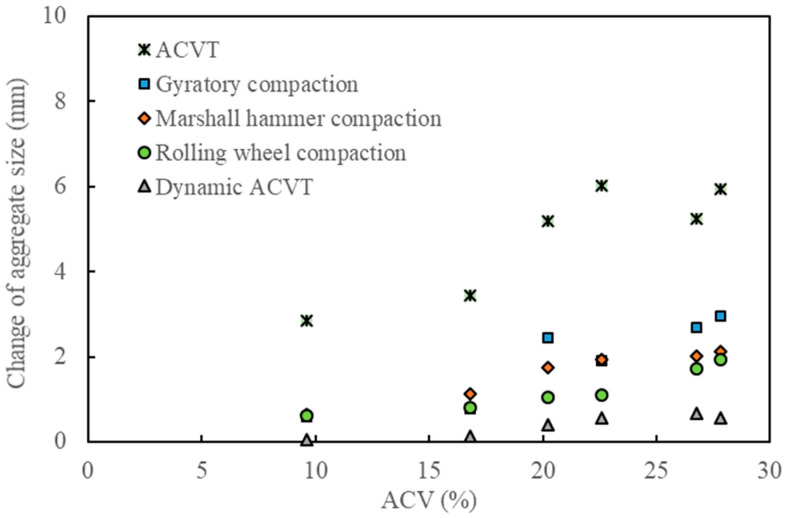
Effect of ACV on the change of aggregate size under various loading methods.

**Figure 14:**
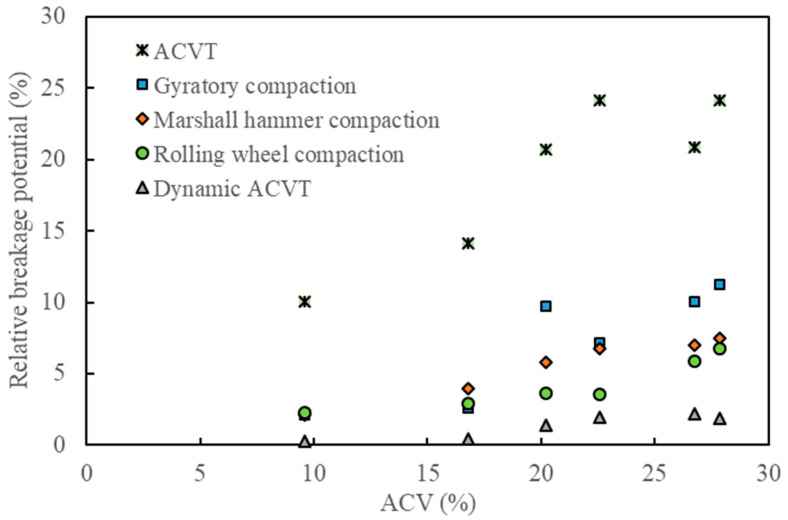
Effect of ACV on the relative breakage potential under various loading methods.

Figure 12, Figure 13 and Figure 14 show the effect of ACV on the breakage ratio, the change of aggregate size and the relative breakage potential under various types of loads. As can be observed from these three figures, standard ACVTs resulted in the most serious aggregate crushing, followed by gyratory compaction, Marshall hammer compaction and roller compaction. Dynamic ACVTs had the slightest effect on aggregate crushing. The effect of ACV seemed to have a critical point at 20%. When ACV was below 20%, the effects of different compaction loads, including gyration, Marshall and roller methods, were close to each other. When ACV was above 20%, the effects of these three compaction methods were significantly varied. Since gyratory shear compaction and Marshall hammer compaction are common compaction modes used for specimen preparation of asphalt concrete in the laboratory, it was seen that gyratory shear compaction tended to result in more aggregate crushing compared to the Marshall impact method. Roller compaction led to less aggregate crushing compared to the gyratory and Marshall methods. Combining the data shown in Figure 12, Figure 13 and Figure 14, it was indicated that the traditional compaction methods can also result in serious aggregate crushing, especially for coarse aggregates with ACV larger than 20%. In this study, the breakage ratio can reach 20~40% in cases where single-size coarse aggregates (9.5–13.2 mm) were tested. The effect of traffic loads, which were simulated by dynamic ACVTs for 100,000 cycles, seemed to be very limited on aggregate crushing. Furthermore, gyratory shear compaction, Marshall impact compaction and roller compaction all have different loading methods; however, their effects on the aggregate crushing mode seemed to be the same for strong aggregates with low ACV. Gyratory shear-induced force can result in more serious aggregate crushing and thus relatively more small and fine fractions compared to Marshall impact compaction and roller compaction.

**Figure 15:**
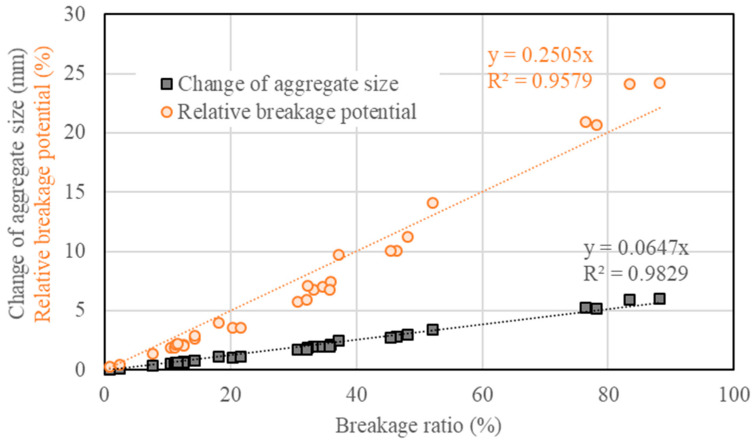
Relations between the breakage ratio, the change of aggregate size ratio and relative breakage potential.

Figure 15 shows the relations between the breakage ratio, the change of aggregate size ratio and relative breakage potential. A linear relation can fit the data obtained from various types of loads well. A higher breakage ratio tended to result in a larger change in aggregate size, as well as relative breakage potential. This implied that the breakage ratio can be used as a good indicator for aggregate crushing resistance. In this case, the breakage ratio should be controlled below 30% to prevent the risk of excessive aggregate crushing during compaction, based on data in Figure 12. Combined with the regression equations, as indicated in Figure 15, the critical value for the change of aggregate size and the relative breakage potential can be determined as 2.0 mm and 7.5%, respectively. The breakage ratio of 30%, the change of aggregate size of 2.0 mm and the relative breakage potential of 7.5% can be used as the critical values for evaluation of aggregate crushing susceptibility.

**Figure 16:**
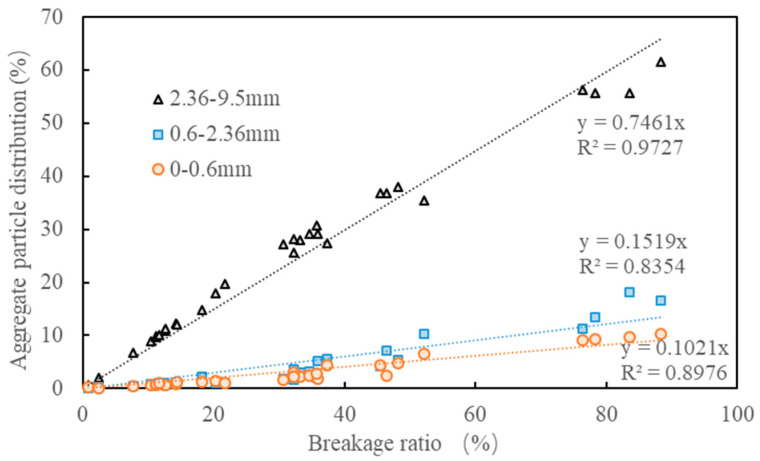
Relations between the breakage ratio and aggregate particle distribution.

Figure 16 shows the relations between the breakage ratio and aggregate particle distribution after being crushed. In total, six types of aggregate and five types of loads were included in Figure 16. The crushed aggregates were divided into coarse fraction (2.36–9.5 mm), small fraction (0.6–2.36 mm) and fine fraction (0–0.6 mm) to investigate the particle distribution characteristics. In general, the content of these three fractions linearly increased with an increased breakage ratio, independent of type of load and aggregate. The linear fitting results indicate that the coarse fraction accounted for 75%, while the content for small and fine fractions was 15% and 10%, respectively. This strongly implied that the contribution of the fracture mode was predominant, while the attrition and abrasion modes remained relatively limited for aggregate crushing under various types of loads.

## 4. Finite Element Modelling

### 4.1. Modelling of Roller Loads

#### 4.1.1. Model Characteristics

Finite element modelling was carried out to investigate the effects of roller loads on the compressive stress level and distribution within asphalt mixtures during construction and compaction. The modelling stress level was thus compared with those stress levels that were applied for dynamic ACVTs. By doing this, the risk of aggregate breakage was evaluated. Heavy vibratory tandem rollers are usually able to generate more compaction force compared to pneumatic tired rollers and thus result in a higher risk of aggregate breakage during compaction. For this reason, a heavy vibratory tandem roller with a working weight of 13 tons was selected for mixture compaction modelling. The steel wheel had a diameter of 1236 mm and a width of 2135 mm. The static line pressure was 300 N/cm and the exciting force was 126 kN. The modelled asphalt pavement consisted of an 80 mm asphalt layer, 180 mm cement stabilized base course and 180 mm cement stabilized sub-base course. The length of the pavement was selected as 2000 mm to reduce the boundary effect. The base course and sub-base course were modelled as linear elastic with an elastic modulus of 15,000 MPa and 14,000 MPa, respectively. It was reported that the dynamic complex modulus of asphalt mixture at a high temperature of 60 °C was in the order between 100 MPa and 1000 MPa [25,26]. As the compaction temperature is usually higher than 135 °C, it can be foreseen that the asphalt mixture modulus at such a high temperature should be in the order of 10 MPa and 100 MPa. For this reason, the asphalt layer was modelled in a wide range of elastic modulus (10–320 MPa) by considering the increasing modulus during compaction. Due to the huge modulus difference between the steel and asphalt mixture, the steel wheel was modelled with a rigid body to limit the mathematical size of the model. The contact properties between the roller wheel and asphalt mixture were selected as hard contact and frictionless. For the purpose of simplification, the model made use of the ABAQUS 2D plate stress model. The boundary condition was as follows: movements at the bottom of the model were fully restrained; at the vertical boundaries of the model, only horizontal movements were restrained.

#### 4.1.2. Modelling Results

**Figure 17:**
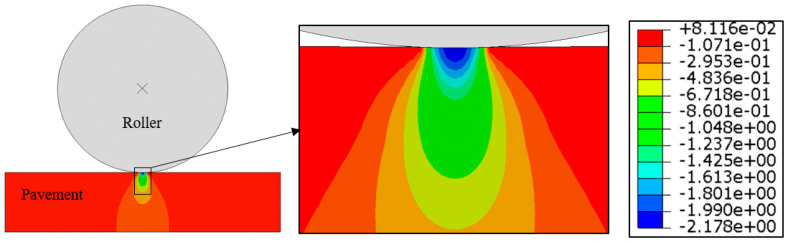
Vertical compressive stress contour under vibratory compaction and an asphalt mixture modulus of 100 MPa.

**Figure 18:**
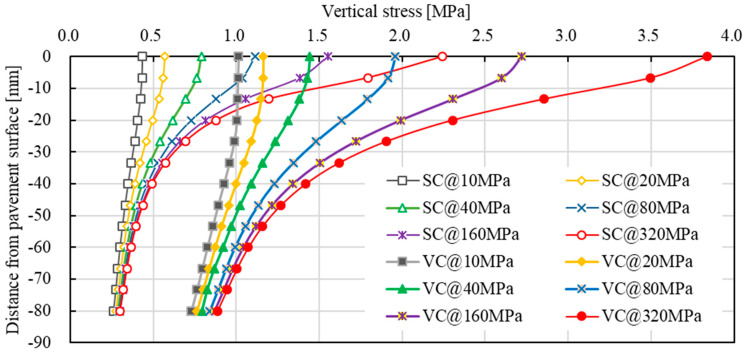
Effects of the asphalt mixture modulus on vertical stress distribution under static and vibratory roller compaction (SC: static compaction; VC: vibratory compaction; 10 MPa, 10 MPa, 20 MPa, 40 MPa, 80 MPa, 160 MPa, 320 MPa: asphalt mixture modulus).

Figure 17 gives the vertical compressive stress contour under vibratory compaction at an asphalt mixture modulus of 100 MPa based on 2D modelling. The selected length and depth of the pavement structure was enough to reduce the effect of the model boundary. The stress contour indicated that high levels of vertical stress happened under the roller–pavement contact region. The vertical stress tended to reduce as the pavement depth increased. Figure 18 shows the detailed results of vertical stress distribution under roller static and vibratory compaction. Effects of the asphalt mixture modulus were also considered by changing the elastic modulus, including 10 MPa, 20 MPa, 40 MPa, 80 MPa, 160 MPa and 320 MPa, respectively. It should be noted that the degree of compaction of the asphalt mixture is increased after each time of roller compaction. This also leads to an increase in the asphalt mixture modulus. As a result, the increased modulus reduces the contact area between roller and pavement and thus a higher vertical stress can be expected. This was well reflected in the modelling results, as indicated in Figure 18. Roller vibratory compaction can lead to a much higher vertical stress compared with static compaction. For instance, the vertical stress level ranged from 1.0 MPa to 3.7 MPa for vibratory compaction, while the corresponding level reduced to a range between 0.4 MPa and 2.3 MPa for static compaction. In this case, the vibratory compaction was very effective in improving the degree of compaction; however, it might be a disadvantage for aggregate crushing resistance. This is especially true for those aggregates with high ACV. Combining the results obtained from dynamic ACVTs, as well as roller compaction, it can be concluded that roller static compaction can be an effective method to prevent aggregate crushing. In the early state of compaction, the modulus was relatively low, the vibratory compaction can be applied to increase the degree of compaction with a relatively low risk for aggregate crushing. Excessive vibratory compaction should be avoided to prevent aggregate crushing, which can well be identified by surface aggregate crushing or breakage. It could be concluded that the stress level induced by the roller load was much lower than that applied for static ACVTs. The application of static ACVTs may lead to an excessive requirement on aggregate crushing resistance, which may be disadvantageous for the application of some certain types of aggregate with high ACV, especially for recycled aggregates obtained from construction and demolition wastes.

### 4.2. Modelling of Marshall Hammer Compaction

#### 4.2.1. Model Characteristic

During the mixture design phase, asphalt mixtures are mainly prepared by Marshall compaction in the laboratory. For dense-graded asphalt mixtures, a compaction effort of 75 blows is commonly given on either side of the specimen, whereas it is only 50 blows in the case of stone mastic asphalt and porous asphalt concrete. Reducing the blow number in these two types of mixtures prevents severe aggregate breakage for higher compaction efforts due to the gap-graded structure and the presence of a strong coarse aggregate skeleton. Aggregate breakage is directly related to the compaction force/stress that the specimen is subjected to. In order to obtain insight into the stress level during specimen preparation using the Marshall blow method, finite element modelling was carried out to simulate the interaction between the specimen and compaction hammer. The model made use of the ABAQUS 3D dynamic explicit model. The model consisted of a compaction hammer with a circular compaction foot (diameter 98.5 mm) and a cylinder specimen (diameter 101.6 mm and height 63.5 mm) of asphalt mixture. The modelled compaction hammer has a 4.536 kg falling mass with a free fall of 457.2 mm. The steel hammer was modelled using a rigid body to limit the mathematical size of the model. The asphalt mixture was modelled as linear elastic in a wide range of modulus (10–320 MPa) by considering the increasing modulus during compaction. The contact properties between hammer and asphalt mixture were selected as hard contact and frictionless. The boundary condition was as follows: the movements at the bottom of asphalt mixture cylinder specimen were fully restrained; at the vertical boundaries of the outer surface, only horizontal movements were restrained. The model was loaded by the gravity of the compaction hammer with a 4.536 kg falling mass and a free fall of 457.2 mm. This led to the hammer impact mixture specimen after falling 0.3055 s with a speed of 2.993 m/s.

#### 4.2.2. Modelling Results

**Figure 19:**
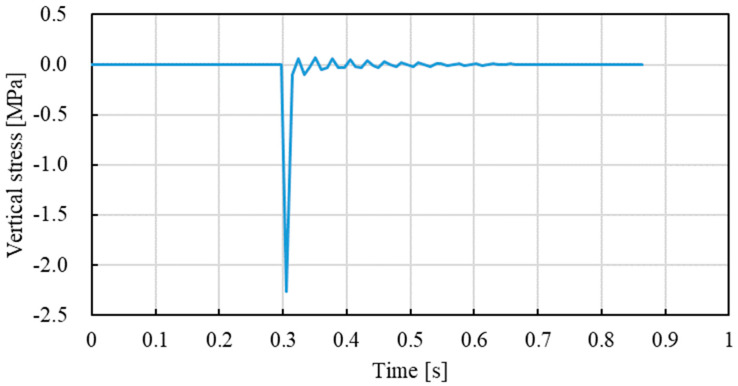
Marshall compaction-induced vertical stress obtained by finite element modelling with an asphalt mixture modulus of 40 MPa.

**Figure 20:**
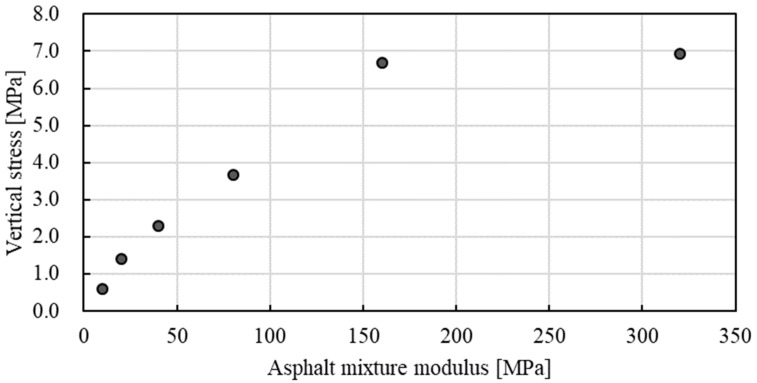
Effect of the asphalt mixture modulus on Marshall compaction-induced vertical stress.

Figure 19 gives the development of vertical stress that the mixture specimen is subjected to over time. As indicated in Figure 19, the model was able to reflect the impact action of the falling hammer on the specimen surface well. The vibration after impact was also able to be captured in the modelling results. Figure 20 gives the effect of the asphalt mixture modulus on Marshall compaction-induced vertical stress. The vertical stress increased linearly when the asphalt mixture modulus increased in the initial state. After the modulus reached 160 MPa, the vertical stress tended to be stable. Further increasing the modulus only led to a slight increase in vertical stress. The final vertical stress can reach a high value of around 7.0 MPa, which is much higher than that obtained by roller vibratory compaction, as shown in Figure 18. It indicates that Marshall compaction may induce higher vertical stress that leads to aggregate breakage compared with real roller compaction. This was demonstrated by the aggregate crushing characteristics between Marshall and roller compaction.

## 5. Conclusions

In this study, various experiments, including static and dynamic aggregate crushing value tests (ACVTs), Marshall hammer compaction tests, gyratory compaction tests and roller compaction tests, were conducted to evaluate the crushing characteristics of six different types of coarse aggregates. Finite element modelling was carried out to investigate the effects of heavy vibratory tandem roller and Marshall hammer compaction loads on compressive stress during the mixture compaction process. Based on the experimental results and the analysis, the following conclusions were drawn:

1. Static ACVTs and dynamic ACVTs with 100,000 loading cycles resulted in a distinct difference in aggregate crushing. Static ACVTs were unable to reflect the crushing behavior of coarse aggregates under traditional Marshall, gyration and roller compaction well.
2. The development of the resilient modulus and accumulative permanent strain under dynamic ACVTs can be well fitted by using a logarithm model and the model parameter showed a strong stress dependency.
3. The type of aggregate had a strong influence on crushing resistance, independent of type of load. When coarse aggregates with high ACV were considered for application, tests should be done to evaluate the risk of aggregate breakage during mixture preparation and construction compaction.
4. The compaction process can be the critical phase for aggregate crushing because of the high breakage ratio, while the risk of aggregate crushing under actual traffic loads may be very limited. The selection of the right compaction methods can be helpful to prevent the risk of excessive aggregate crushing.
5. Marshall, gyration and roller compaction was proved to be capable of evaluating the aggregate crushing resistance by simulating the process of mixture compaction and can distinguish coarse aggregates with high crushing susceptibility well.
6. The aggregate crushing resistance can be evaluated by using ACV, breakage ratio, change of aggregate size and relative breakage potential. It was found that an ACV of 20%, a breakage ratio of 30%, a change of aggregate size of 2.0 mm and a relative breakage potential of 7.5% were the corresponding critical values for aggregate crushing susceptibility.
7. Gyration, Marshall and roller compaction had different loading methods, but they showed a similar fracture mode on aggregate crushing. Gyration compaction induced shear force and resulted in more serious aggregate crushing when compared to the other two compaction methods.
8. The stress levels obtained from finite element modelling on roller compaction and Marshall hammer compaction explained the aggregate crushing results under laboratory roller and Marshall compaction well.
9. The aggregate particle distribution exhibited a linear relation with the breakage ratio. The coarse fraction accounted for 75%, while the small and fine fractions were limited to 15% and 10%, independent of type of load. This indicated that the crushing mechanism was controlled by the fracture mode and the contribution of the attrition and abrasion modes was relatively small.

The application of coarse aggregates with high ACV has to deal with the problem of aggregate crushing during laboratory specimen compaction and field construction. The selection of the proper compaction method and the optimization of the aggregate skeleton may be of importance to prevent excessive aggregate crushing. The traditional dense asphalt mixture structure and the presence of a higher concentration of fine aggregates may be helpful to reduce the breakdown of the aggregate skeleton during compaction. This study was limited to the investigation of coarse aggregates of a single size between 9.5 mm and 13.2 mm. The combination of different sizes of coarse aggregates should be involved in future work. Furthermore, the effect of the addition of an asphalt binder, fine aggregate and filler should be examined. Tests on asphalt mixture should be conducted for the purpose of validation.

## Figures and Tables

**Table 1:** Basic properties of different types of aggregate.

Major Indexes	Type of Aggregate					
	Basalt Aggregate	Steel Slag Aggregate	Limestone Aggregate	Marble Aggregate	Recycled Concrete Aggregate	Slightly Weathered Limestone Aggregate
Apparent relative density/g/cm^3^	2.896	3.601	2.734	2.724	2.689	2.724
Water absorption/%	0.5	1.6	0.6	0.6	2.0	0.7
Aggregate crushing value (ACV)/%	9.6	16.8	20.2	22.6	26.7	27.8
Polished stone value	55	50	37	36	34	35

**Table 2:** Aggregate particle distribution after static ACVTs for various types of aggregate.

Type of Aggregate	9.5–13.2 mm	4.75–9.5 mm	2.36–4.75 mm	1.18–2.36 mm	0.6–1.18 mm	0–0.6 mm
Basalt aggregate	53.54	28.57	8.30	3.35	3.83	2.42
Steel slag	47.80	29.90	5.50	5.30	5.00	6.50
Limestone aggregate	23.57	35.33	20.89	5.06	6.12	9.03
Marble aggregate	21.80	41.75	13.87	8.17	5.21	9.20
Recycled concrete aggregate	11.75	43.38	18.13	9.24	7.25	10.25
Slightly weathered limestone aggregate	16.50	35.30	20.38	10.68	7.38	9.77

**Table 3:** Results of aggregate crushing characteristics under static ACVTs.

Type of Aggregate	Breakage Ratio	ACV	$d^{\prime }$	Δd	RBP	RBP-cf	RBP-sf	RBP-ff
Basalt aggregate	46.46	9.59	8.51	2.84	10.08	7.90	1.68	0.50
Steel slag	52.20	16.80	7.91	3.44	14.13	9.60	3.18	1.35
Limestone aggregate	78.20	22.58	6.16	5.19	20.70	14.63	4.16	1.91
Marble aggregate	88.25	26.74	5.33	6.02	24.19	17.12	4.94	2.13
Recycled concrete aggregate	76.43	20.21	6.11	5.24	20.89	14.93	4.08	1.88
Slightly weathered limestone aggregate	83.50	27.82	5.40	5.95	24.13	17.17	4.93	2.03

Note: RBP = Relative breakage potential (0.075–13.2 mm); RBP-cf = Relative breakage potential of coarse fraction (2.36–13.2 mm); RBP-sf = Relative breakage potential of small fraction (0.6–2.36 mm); RBP-ff = Relative breakage potential of fine fraction (0.075–0.6 mm).

**Table 4:** Fitting results of model parameters for the development of resilient modulus and the accumulative permanent strain.

Type of Aggregate	Stress Level	Modulus Fitting			Permanent Strain Fitting	
		A	E_0_	R^2^	C	R^2^
Basalt aggregate	0.7 MPa	1.15	139	0.935	0.050	0.991
	1.4 MPa	1.33	160	0.958	0.089	1.000
	2.1 MPa	1.62	191	0.950	0.103	0.996
Steel slag	0.7 MPa	1.67	127	0.946	0.099	0.992
	1.4 MPa	1.98	153	0.981	0.158	1.000
	2.1 MPa	2.10	179	0.958	0.192	0.989
Limestone aggregate	0.7 MPa	2.81	121	0.949	0.147	0.995
	1.4 MPa	2.95	139	0.981	0.176	1.000
	2.1 MPa	3.15	168	0.892	0.269	0.987
Marble aggregate	0.7 MPa	2.28	118	0.852	0.149	0.985
	1.4 MPa	2.43	135	0.983	0.235	1.000
	2.1 MPa	2.60	166	0.935	0.290	0.984
Recycled concrete aggregate	0.7 MPa	2.56	113	0.970	0.135	0.993
	1.4 MPa	3.16	134	0.990	0.195	0.994
	2.1 MPa	3.56	152	0.992	0.275	0.993
Slightly weathered limestone aggregate	0.7 MPa	3.03	109	0.964	0.245	0.993
	1.4 MPa	3.85	125	0.985	0.335	0.994
	2.1 MPa	4.12	160	0.948	0.395	0.993

**Table 5:** Aggregate particle distribution after dynamic ACVTs.

Type of Aggregate	Stress Level	9.5–13.2 mm	4.75–9.5 mm	2.36–4.75 mm	1.18–2.36 mm	0.6–1.18 mm	0–0.6 mm
Basalt aggregate	0.7 MPa	99.78	0.11	0.02	0.01	0.01	0.07
	1.4 MPa	99.55	0.27	0.04	0.02	0.02	0.10
	2.1 MPa	99.16	0.47	0.10	0.04	0.03	0.20
Steel slag	0.7 MPa	99.32	0.46	0.08	0.04	0.02	0.08
	1.4 MPa	98.66	0.97	0.12	0.08	0.05	0.12
	2.1 MPa	97.57	1.81	0.27	0.14	0.06	0.15
Limestone aggregate	0.7 MPa	97.98	1.60	0.18	0.05	0.05	0.14
	1.4 MPa	95.00	3.71	0.73	0.11	0.22	0.23
	2.1 MPa	92.37	5.76	0.98	0.16	0.33	0.39
Marble aggregate	0.7 MPa	97.67	1.61	0.29	0.09	0.17	0.17
	1.4 MPa	92.94	5.28	0.89	0.23	0.30	0.36
	2.1 MPa	89.58	7.77	1.21	0.44	0.45	0.55
Recycled concrete aggregate	0.7 MPa	96.60	2.40	0.43	0.17	0.18	0.22
	1.4 MPa	90.41	7.28	0.83	0.52	0.54	0.42
	2.1 MPa	87.46	9.92	0.95	0.55	0.55	0.56
Slightly weathered limestone aggregate	0.7 MPa	97.37	1.86	0.24	0.17	0.16	0.20
	1.4 MPa	91.40	6.95	0.55	0.31	0.47	0.32
	2.1 MPa	88.80	9.10	0.77	0.51	0.25	0.58

**Table 6:** Results of aggregate crushing characteristics under dynamic ACVTs.

Type of Aggregate	Stress Level	Breakage Ratio	$d^{\prime }$	Δd	RBP	RBP-cf	RBP-sf	RBP-ff
Basalt aggregate	0.7 MPa	0.22	11.33	0.02	0.08	0.04	0.02	0.01
	1.4 MPa	0.45	11.32	0.03	0.13	0.08	0.03	0.02
	2.1 MPa	0.84	11.29	0.06	0.26	0.16	0.06	0.04
Steel slag	0.7 MPa	0.68	11.31	0.04	0.16	0.11	0.03	0.02
	1.4 MPa	1.34	11.27	0.08	0.28	0.21	0.05	0.02
	2.1 MPa	2.43	11.22	0.13	0.45	0.36	0.06	0.03
Limestone aggregate	0.7 MPa	2.02	11.24	0.11	0.36	0.28	0.05	0.03
	1.4 MPa	5.00	11.08	0.27	0.89	0.73	0.12	0.05
	2.1 MPa	7.63	10.94	0.41	1.37	1.10	0.19	0.08
Marble aggregate	0.7 MPa	2.33	11.21	0.14	0.49	0.37	0.09	0.04
	1.4 MPa	7.06	10.96	0.39	1.28	1.03	0.18	0.07
	2.1 MPa	10.42	10.78	0.57	1.92	1.53	0.27	0.11
Recycled concrete aggregate	0.7 MPa	3.40	11.16	0.19	0.68	0.53	0.11	0.05
	1.4 MPa	9.59	10.83	0.52	1.75	1.40	0.26	0.09
	2.1 MPa	12.54	10.68	0.67	2.18	1.76	0.31	0.12
Slightly weathered limestone aggregate	0.7 MPa	2.63	11.20	0.15	0.56	0.41	0.10	0.04
	1.4 MPa	8.60	10.90	0.45	1.46	1.18	0.21	0.07
	2.1 MPa	11.20	10.77	0.58	1.89	1.53	0.24	0.12

**Table 7:** Aggregate particle distribution after Marshal hammer compaction.

Type of Aggregate	9.5–13.2 mm	4.75–9.5 mm	2.36–4.75 mm	1.18–2.36 mm	0.6–1.18 mm	0–0.6 mm
Basalt aggregate	87.38	10.57	0.77	0.35	0.26	0.69
Steel slag	81.86	10.72	4.06	1.28	0.90	1.19
Limestone aggregate	69.32	20.81	6.36	1.12	0.71	1.69
Marble aggregate	66.84	22.13	5.77	1.90	1.08	2.28
Recycled concrete aggregate	65.38	23.47	5.62	1.83	1.34	2.37
Slightly weathered limestone aggregate	64.05	23.64	5.42	2.30	2.83	1.76

**Table 8:** Results of aggregate crushing characteristics after Marshal hammer compaction.

Type of Aggregate	Breakage Ratio	$d^{\prime }$	Δd	RBP	RBP-cf	RBP-sf	RBP-ff
Basalt aggregate	12.62	10.71	0.64	2.07	1.66	0.26	0.14
Steel slag	18.14	10.23	1.12	3.97	3.12	0.60	0.25
Limestone aggregate	30.68	9.61	1.74	5.79	4.75	0.69	0.35
Marble aggregate	33.16	9.42	1.93	6.74	5.29	0.98	0.47
Recycled concrete aggregate	34.62	9.34	2.01	7.01	5.47	1.05	0.49
Slightly weathered limestone aggregate	35.95	9.22	2.13	7.45	5.86	1.22	0.37

**Table 9:** Aggregate particle distribution after gyratory compaction.

Type of Aggregate	9.5–13.2 mm	4.75–9.5 mm	2.36–4.75 mm	1.18–2.36 mm	0.6–1.18 mm	0–0.6 mm
Basalt aggregate	88.76	8.87	0.79	0.34	0.34	0.90
Steel slag	85.76	10.99	1.29	0.62	0.43	0.91
Limestone aggregate	62.76	19.70	7.74	3.00	2.46	4.35
Marble aggregate	67.81	21.49	4.19	1.94	1.56	3.01
Recycled concrete aggregate	54.57	30.26	6.63	1.75	2.43	4.36
Slightly weathered limestone aggregate	51.80	29.70	8.30	2.70	2.70	4.80

**Table 10:** Results of aggregate crushing characteristics after gyratory compaction.

Type of Aggregate	Breakage Ratio	$d^{\prime }$	Δd	RBP	RBP-cf	RBP-sf	RBP-ff
Basalt aggregate	11.24	10.75	0.60	2.11	1.59	0.34	0.19
Steel slag	14.24	10.58	0.77	2.61	2.04	0.38	0.19
Limestone aggregate	37.24	8.89	2.46	9.73	6.93	1.90	0.90
Marble aggregate	32.19	9.43	1.92	7.13	5.23	1.28	0.63
Recycled concrete aggregate	45.43	8.65	2.70	10.07	7.35	1.82	0.91
Slightly weathered limestone aggregate	48.20	8.38	2.97	11.26	8.21	2.06	1.00

**Table 11:** Aggregate particle distribution after roller compaction.

Type of Aggregate	9.5–13.2 mm	4.75–9.5 mm	2.36–4.75 mm	1.18–2.36 mm	0.6–1.18 mm	0–0.6 mm
Basalt aggregate	88.33	9.05	0.95	0.32	0.30	1.05
Steel slag	85.71	10.49	1.52	0.52	0.55	1.21
Limestone aggregate	79.67	16.61	1.25	0.46	0.61	1.40
Marble aggregate	78.31	18.00	1.65	0.46	0.48	1.10
Recycled concrete aggregate	67.85	25.24	2.87	0.75	0.97	2.31
Slightly weathered limestone aggregate	64.30	27.80	3.00	0.80	1.30	2.80

**Table 12:** Results of aggregate crushing characteristics after roller compaction.

Type of Aggregate	Breakage Ratio	$d^{\prime }$	Δd	RBP	RBP-cf	RBP-sf	RBP-ff
Basalt aggregate	11.67	10.72	0.63	2.26	1.67	0.37	0.22
Steel slag	14.29	10.55	0.80	2.88	2.15	0.48	0.25
Limestone aggregate	20.33	10.29	1.06	3.60	2.77	0.54	0.29
Marble aggregate	21.69	10.25	1.10	3.53	2.87	0.43	0.23
Recycled concrete aggregate	32.15	9.63	1.72	5.89	4.52	0.88	0.48
Slightly weathered limestone aggregate	35.70	9.42	1.93	6.76	5.09	1.09	0.58

## Data Availability

No relevant.
